# Adult pancreatoblastoma: clinical features and Imaging findings

**DOI:** 10.1038/s41598-020-68083-2

**Published:** 2020-07-09

**Authors:** Xi Zhang, Shu-juan Ni, Xiao-hong Wang, Dan Huang, Wei Tang

**Affiliations:** 1 Department of Radiology, Fudan University Shanghai Cancer Center, 270 Dongan Road, Shanghai, 200032 China; 2 Department of Pathology, Fudan University Shanghai Cancer Center, Shanghai, China; 3 Department of Oncology, Shanghai Medical College of Fudan University, Shanghai, China

**Keywords:** Cancer imaging, Gastrointestinal cancer

## Abstract

The objective of this study was to illustrate the clinical, CT, MRI, and ^18^F-FDG PET/CT features of adult pancreatoblastoma, an extremely rare disease. In this study, the clinical and imaging features of seven adult patients with pathologically confirmed pancreatoblastoma were retrospectively analyzed. The following parameters were evaluated: size, location, shape, margination, solid-cystic ratio, CT attenuation values or signal intensity and contrast enhancement pattern. We also analyzed whether abnormal FDG uptake occurred during ^18^F-FDG PET/CT imaging. All seven patients were male (mean age 45 years; range 22–65 years). Six tumors were irregular in shape, exogenous, and grew outward from the pancreatic parenchyma, similar to branches growing from a tree trunk (85.7%). The tumor margins were clear in five patients (71.4%), and three tumors (42.9%) were encapsulated. Six tumors (71.4%) were solid, with homogeneous enhancement observed on contrast-enhanced CT and MRI. Dynamic-enhanced CT and MRI showed progressive enhancement for all tumors. On ^18^F-FDG PET/CT, one tumor exhibited abnormal FDG uptake, and two tumors exhibited no abnormal uptake (66.7%). In conclusion, adult pancreatoblastoma most commonly occurs in male patients, and it usually appears as an exophytic, irregular, and hypovascular mass with well-defined margins and progressive enhancement on CT and MRI. This type of tumor always grows out of the parenchyma of the pancreas, similar to branches growing outward from a tree trunk.

## Introduction

Pancreatoblastoma (PB) is a very rare malignant epithelial neoplasm of the pancreas with multiple lines of differentiation. The annual incidence of PB is approximately 0.004 per 100,000 people, and it afflicts individuals of all ages^1^. The vast majority of PB has been detected in children, with a median age of 5 years at presentation^2^. This tumor type is more common in Asians, and two-thirds of the tumors in the medical literature have presented in patients of Asian descent^3–5^. More rarely, PB can occur in adults, and the prognosis is worse for adults than for children^5–9^. Therefore, it has been suggested that this tumor type shows biological differences between these two age groups^9^.

Currently, fewer than 50 adult PB cases have been reported in the literature^10^, and the information on these patients is mainly limited to clinical and pathological characteristics. To the best of our knowledge, a comprehensive analysis of their radiological features has not yet been reported. In this article, we present the cases of seven adult patients with pathology-proven PB who were examined at our institution and characterize the imaging appearance of these extremely rare tumors.

## Materials and methods

### Patients

The Institutional Review Board of Fudan University Shanghai Cancer Center approved this retrospective study and waived the requirement for informed consent. All experiments and methods were performed in accordance with relevant guidelines and regulations. Between January 2010 and December 2018, seven adult patients with a pathological diagnosis of PB after surgical resection were retrospectively identified. All seven patients were male [mean age 45 ± 15.55 years (range 22–65 years)]. The symptoms included epigastric pain (n = 3), weight loss (n = 1), and bitter taste (n = 1). Three patients were asymptomatic, and their tumors were discovered incidentally.

Of the seven patients, five underwent a triphasic CT scan [unenhanced, arterial phase (AP) and venous phase (VP)], and two underwent a quadriphasic CT scan [unenhanced, AP, portal venous phase (PVP) and VP]. Dynamic contrast-enhanced MRI was performed for two patients, and ^18^F-fluorodeoxyglucose positron emission tomography/CT (^18^F-FDG PET/CT) was performed on three patients.

### Image acquisition via CT

CT imaging for the seven patients was performed using different scanners (SOMATOM Sensation 64, SOMATOM Definition AS+, Siemens Medical Solutions, Business Unit CT, Forchheim, Germany). Additionally, 300 mg of iodine per milliliter of nonionic contrast material (Ultravist 300; Schering, Berlin, Germany) was administered using a power injector (Ulrich Medizintechnik, Buchbrunnenweg, Germany) through an 18-gauge intravenous catheter placed in the antecubital vein. A fixed dose of 1.5 ml of contrast agent per kilogram of body weight was administered at a rate of 3 ml/s. A computer-assisted bolus-tracking technique was used to determine the optimal scanning delay of the AP, which started 30 s after automatically detecting the peak aortic enhancement. The PVP and VP were scanned 60 and 80 s after the start of contrast material injection, respectively. The parameters for the CT examinations were as follows: tube voltage, 120 kV; tube current, 250 mA; and slice thickness, 3 mm or 1 mm.

### Image acquisition via MRI

A total of two patients underwent 3.0 T MRI scans (Signa; GE Medical Systems). The MRI sequences included T1-weighted in-phase (TR: 220, TE: 2.44), opposed-phase (TR: 220, TE: 5.8), and fat-saturated T2-weighted axial fast spin echo (FSE) (TR: 6,315.8, TE: 86.3) sequences. After intravenous administration of gadopentetate dimeglumine at a dose of 0.1 mmol/kg body weight (Magnevist; Bayer Schering Pharma AG, Berlin, Germany), dynamic contrast-enhanced images were acquired using liver acquisition with a volume acceleration (LAVA) sequence (TR: 2.54, TE: 1.168). The delay time was 20 s for the arterial phase, 60 s for the portal venous phase, and 120 s for coronal delayed phase sequences.

### Image acquisition via ^18^F-FDG PET/CT

Two patients underwent ^18^F-FDG PET/CT examinations at our hospital, and another patient underwent an ^18^F-FDG PET/CT examination at a different hospital. All patients were requested to fast at least 4 h before the ^18^F-FDG PET/CT scan. At the time of tracer injection (dose: 7.4 MBq/kg), the serum glucose level should be controlled between 3.9 mmol/l and 7.1 mmol/l. Before and after injection, patients were kept lying comfortably in a quiet, dimly lit room. Scanning was initiated 1 h after administration of the tracer. The images were obtained on a Siemens Biograph 16HR PET/CT scanner (Siemens, Munich, Germany). The transaxial intrinsic spatial resolution was 4.1 mm (full-width at half-maximum) in the center of the field of view. The data acquisition procedure was as follows: CT scanning was first performed from the proximal thighs to the head (120 kV, 80,250 mA, pitch 3.6, and rotation time 0.5). Immediately after CT scanning, PET emission scanning that covered the identical transverse field of view was performed. The acquisition time was 2.3 min per table position. PET imaging data sets were reconstructed iteratively by applying the CT data for attenuation correction, and coregistered images were displayed on a workstation^11^.

### Image analysis

This retrospective evaluation was performed on a PACS workstation (GE Centricity PACS V 3.0, GE Healthcare, Milwaukee, WI, USA). The AW Server 2.0 reconstruction software was integrated into the PACS system. Multiplanar reformation (MPR) was performed on all CT images to observe the shape of the lesion and to measure its maximum diameter.

CT and MRI images were independently evaluated by two radiologists [with 10 years (W.T.) and 20 years (X.H.W.) of experience in abdominal radiology] who were blinded to the pathological results. If there were inconsistencies in the categorical variables, a consensus was achieved through discussion. The evaluated parameters included the following: tumor location, size, and shape (round, oval, or irregular); presence of calcification; tumor encapsulation; presence of intrapancreatic or exogenous tumors; solid-to-cystic component ratio; tumor margin (sharp or indistinct); attenuation/intensity (hypo-, iso-, or hyperattenuation/intensity relative to the normal pancreas) in the nonenhanced phase; dilatation of the pancreatic duct (4 mm) or common bile duct (10 mm); parenchymal atrophy of the pancreas; enhancement pattern; metastasis; and invasion of major vessels and adjacent organs.

The solid-to-cystic component ratio can be classified as purely solid (solid components > 90%), mixed solid and cystic, and purely cystic (cystic components > 90%).

The CT attenuation value of the PB was also assessed. The CT attenuation value in Hounsfield units was obtained using a region of interest (ROI) analysis. Three ROIs were also identified in the normal pancreatic parenchyma adjacent to the tumor, and the CT attenuation value of the surrounding normal pancreatic parenchyma was calculated as the mean of these three ROI values. When defining ROIs, special attention was focused on excluding cystic areas, calcification, the pancreatic duct, and the surrounding vessels. Time-density curves (TACs) were drawn on the basis of each CT attenuation value.

On ^18^F-FDG PET/CT, the lesions were determined to be positive by visual analysis if they exhibited higher uptake than adjacent normal tissues. Semiquantitative measurements were represented by the maximum standardized uptake value (SUVmax), which was defined as the ratio of activity per milliliter of tissue to the activity of the injected dose for PET/CT imaging corrected by decay and by the patient’s body weight.

### Histopathological analysis

All resected specimens in this study were reviewed by two gastrointestinal pathologists [with 6 years (S.J.N.) and 10 years (D.H.)] of experience in gastrointestinal pathology to confirm PB and the presence of tumor encapsulation, lymph node metastases, invasion of visceral vessels, and involvement of the surrounding organs.

### Statistical analysis

Quantitative data with a normal distribution are presented as the mean ± standard deviation, and categorical data are expressed as numbers (percentages). The CT attenuation values were compared between each phase by using one-way analysis of variance (ANOVA). The CT attenuation values of the tumor and pancreatic parenchyma in each phase were compared by two-sample paired t-tests. Data were analyzed using IBM SPSS 20 (SPSS, Chicago, IL, USA), and p values less than 0.05 were considered statistically significant.

## Results

The clinical and imaging features of the seven patients are listed in Table 1.

**Table 1:** Clinical and imaging features of the seven adult patients with PB.

Patient no.	1	2	3	4	5	6	7
Age/sex	55/M	37/M	36/M	37/M	22/M	65/M	61/M
Symptom	Bitter taste	Abdominal pain	Abdominal pain	Abdominal pain	None	None	None
AFP (0–10 ng/ml)	2.87	7.4	5.55	3.1	> 3,630	2.13	7.62
CA19-9 (0–27 U/ml)	8.39	6.16	34.82	6.25	1.2	6.07	11.7
Radiological studies	MRI, PET/CT, CT	CT	CT, MRI, PET/CT	CT	CT,	CT, PET/CT	CT
Location	Head	Tail	Head	Head	Body	Neck	Tail
Shape	Irregular	Irregular	Irregular	Irregular	Irregular	Irregular	Round
Exophytic growth	Yes	Yes	Yes	Yes	Yes	Yes	Yes
Size (cm)	2.3 × 4.3	3.1 × 4.7	3.1 × 5.5	5.0 × 6.6	7.3 × 12.4	3.6 × 5.0	4.3 × 5.6
Cystic or necrotic component	Purely solid	Purely solid	Purely solid	Purely solid	Solid and cystic	Purely solid	Purely solid
Border and margin	Partly indistinct	Sharp	Indistinct	Sharp	Sharp	Sharp	Sharp
Encapsulation	No	Yes	No	Yes	Yes	No	No
Calcification	No	Yes	No	No	No	No	No
Pancreatic duct obstruction	No	No	Yes	No	No	No	No
Hemorrhage	No	No	No	No	Yes	No	No
Unenhanced CT	Hypo	Iso	Hyper	Hypo	Hypo	Iso	ISO
CT AP	Hypo	Hypo	Hypo	Hypo	Hypo	Hypo	Hypo
CT PVP	–	–	Hypo	–	Hypo	–	–
CT VP	Hypo	Hypo	Hypo	Hyper	Hypo	Iso	Iso
Tumor homogeneity	Yes	Yes	Yes	Yes	No	Yes	Yes
T1WI	Iso	–	Iso	–	–	–	–
T2WI	Hyper	–	Iso	–	–	–	–
18F-PET/CT	No uptake	–	Uptake	–	–	No uptake	–
Invasion of adjacent structures	Yes	No	No	No	No	No	No
Metastases	Liver	No	No	No	No	No	No

*Hypo* hypoattenuation, relative to the normal pancreas, *iso* isoattenuation, relative to the normal pancreas, *hyper* hyperattenuation, relative to the normal pancreas.

### Preoperative diagnosis

**Figure 1:**
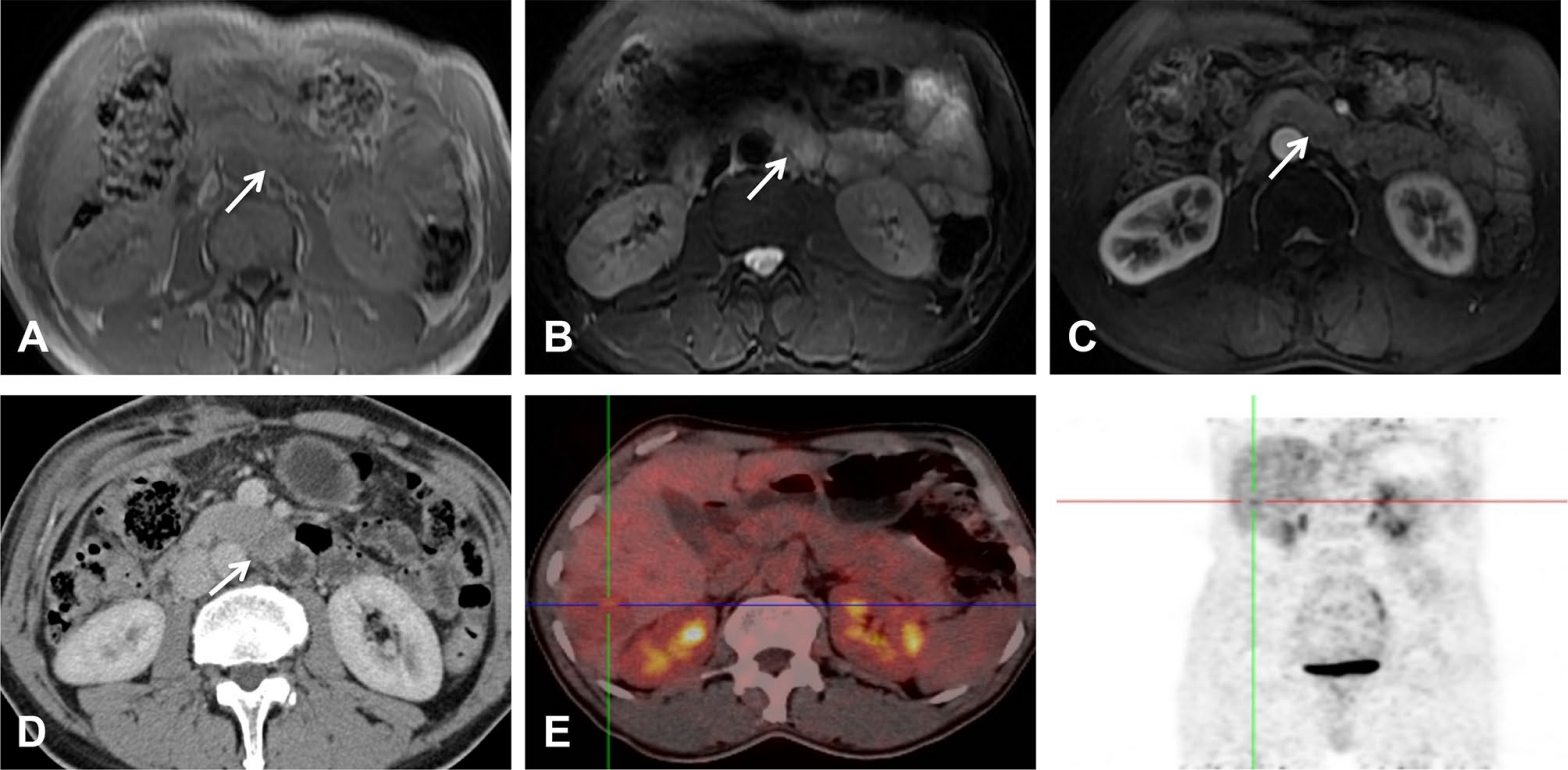
MRI, CT and ^18^F-FDG PET/CT images of a 37-year-old man with PB and multiple liver metastases. The exogenous mass in the uncinate process of the pancreas was isointense on T1WI (**A**) and hyperintense on axial fat-suppressed T2WI (**B**). In the AP, the enhancement of the tumors was lower than that of the surrounding normal pancreas (**C**). The above imaging findings of the tumors are similar to those of the surrounding intestines. On the PET/CT image (**E**), there was no radioactive uptake in the pancreatic tumor, and only one of the multiple metastatic lesions in the liver showed focal uptake (cross-hairs). (**D**) One month after the MRI scan, the CT scan revealed a cylindrical exophytic neoplasm of the uncinate process of the pancreas, similar to branches growing outward from a tree trunk.

The initial reported imaging diagnosis was a hepatic malignant tumor in one patient (case 1), malignant tumors of the pancreas in two patients (cases 3 and 4), pancreatic acinar cell carcinoma (ACC) and solid pseudopapillary neoplasm (SPN) of the pancreas in one patient each (cases 5 and 7), and neuroendocrine neoplasm in two patients (cases 2 and 6). In the patient initially diagnosed with hepatic malignant tumors (case 1), only MRI examination was performed prior to local resection of multiple liver lesions. MRI revealed multiple tumors in the liver, while the exogenous tumor in the uncinate process of the pancreas was located almost completely outside the pancreas; additionally, its signal and morphology could not be distinguished from that of the small intestine (Fig. 1). Furthermore, in the whole-body ^18^F-FDG PET/CT examinations, contrast was not taken up by the pancreatic lesion, and consequently, this pancreatic lesion was missed before surgery.

### Imaging features

**Figure 2:**
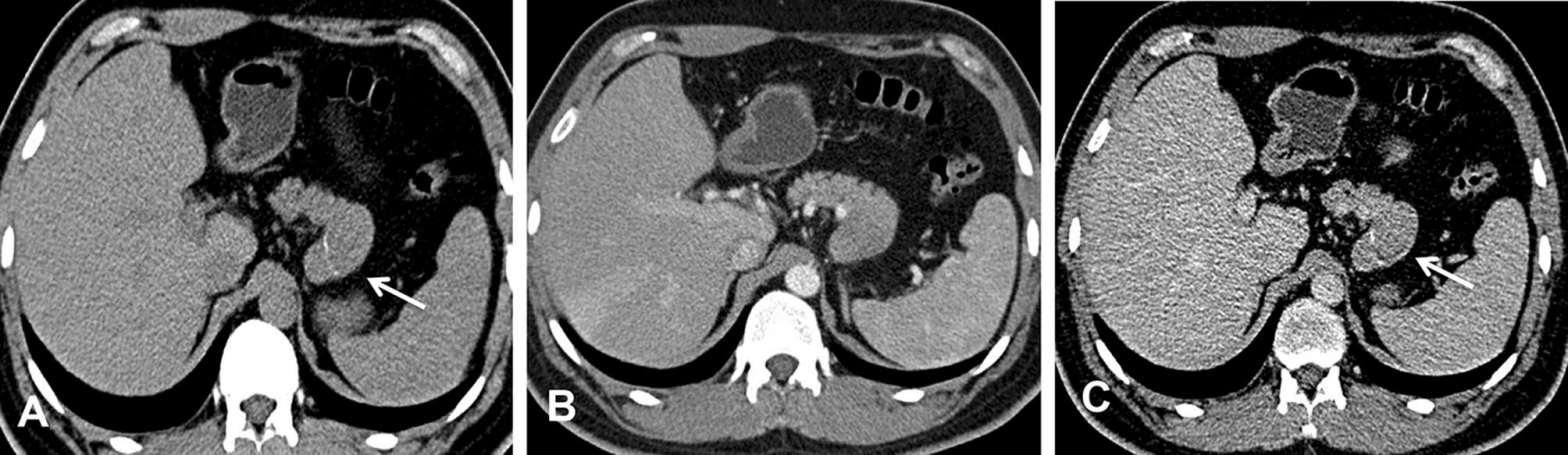
CT images of a 36-year-old man with surgically confirmed PB. (**A**) Nonenhanced CT scan revealed an exogenous mass with calcification in the tail of the pancreas (arrow). (**B**) The tumor exhibits an exophytic growth pattern, similar to branches growing outward from a tree trunk. In the AP, the tumor showed uniform enhancement, but the degree of enhancement was lower than that of the normal pancreatic parenchyma. (**C**) In the VP, the tumor presented as a well-defined mass with enhanced encapsulation (arrow) that had slightly less enhancement than the normal pancreatic parenchyma.

**Figure 3:**
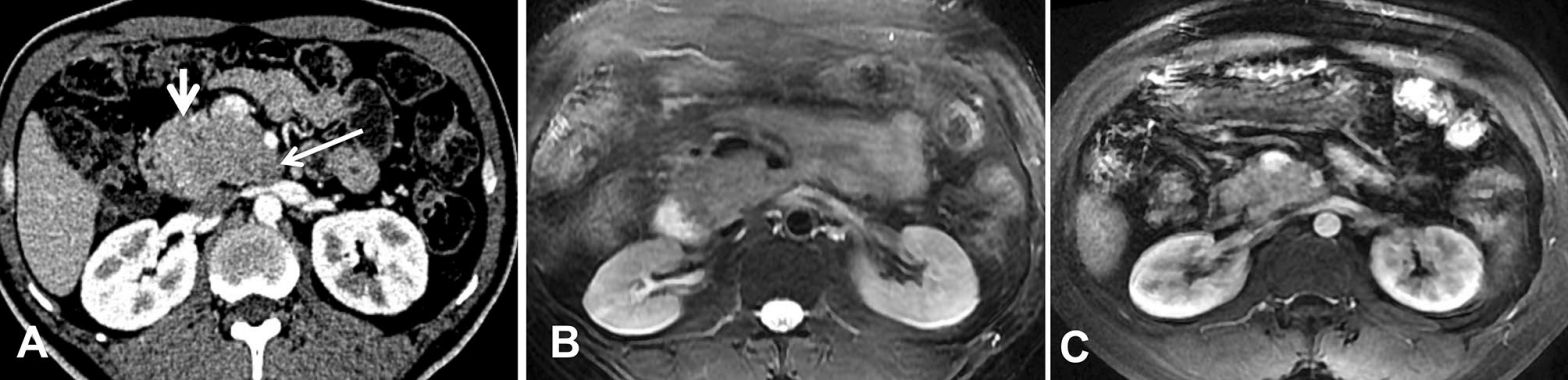
CT and MRI images of a 36-year-old man with pathologically confirmed PB. (**A**) In the AP view, the tumors presented as exogenous hypovascular masses in the head of the pancreas. The tumor exhibits an exophytic growth pattern, similar to branches growing outward from a tree trunk, and the long arrows indicate that the tumors have a tapering head. The short arrows indicate that the interface between the root of the tumors and the head of the pancreas is not clear. The MRI examination showed that the mass was isointense on T2WI (**C**).

**Figure 4:**
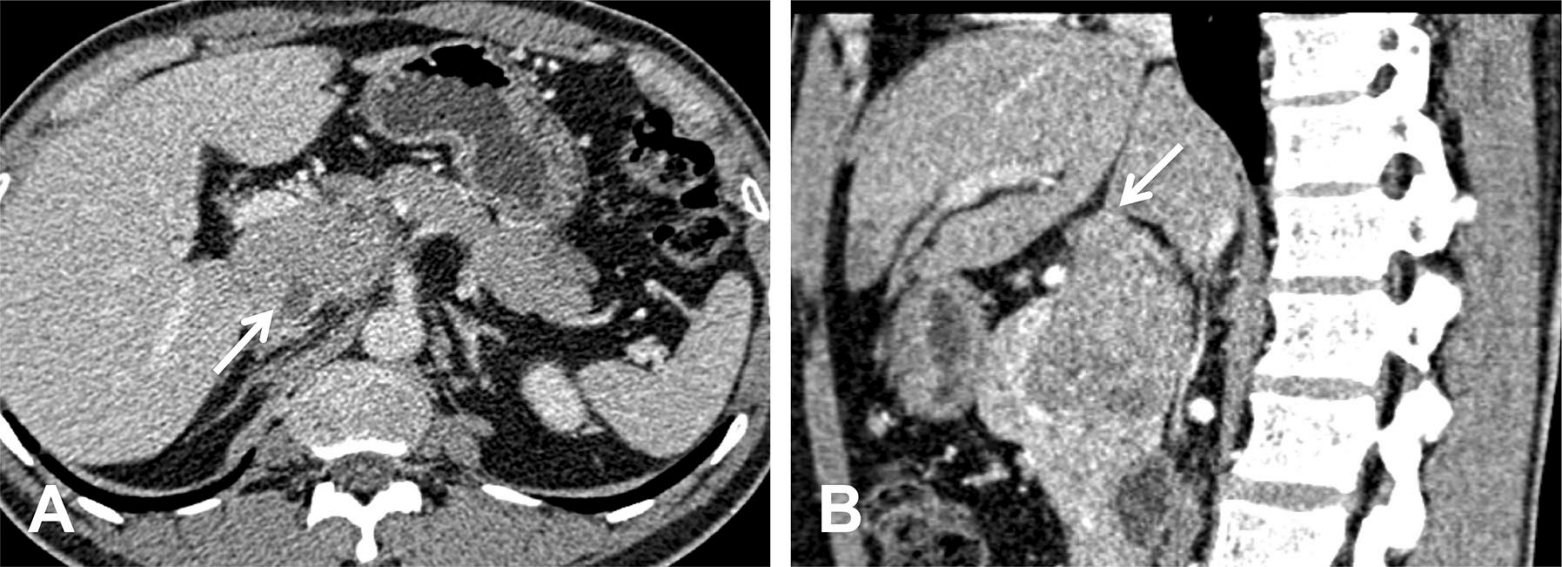
CT image of a 37-year-old man with pathologically confirmed PB. (**A**) In the VP view, the mass appears as an isodense mass with well-defined cystic degeneration in the peripheral portion of the mass. (**B**) The sagittal MPR images show that the tumor exhibits an exophytic growth pattern, similar to branches growing outward from a tree trunk, with a pointed head deep into the hilum of the liver (arrow).

In our study, the average tumor dimension was 4.1 × 6.3 cm (range 2.3 × 4.3 to 7.3 × 12.4 cm). Tumors were located in the head of the pancreas in three patients (42.9%), in the tail of the pancreas in two patients, and in the neck and body of the pancreas in one patient. On the MPR images, one of the lesions appeared round, and the other six (85.7%) appeared irregular in shape. All seven tumors were exogenous (100%), and six tumors (85.7%) grew outward from the pancreatic parenchyma, similar to branches growing from a tree trunk (Figs. 1D, 2C, 3A, 4B). Two of the tumors located in the head and neck of the pancreas showed a tapering head that extended into the hilum of the liver (Fig. 4B). In total, five tumors (71.4%) showed distinct margins (Fig. 1), and two tumors (28.6%) showed partly distinct or indistinct margins (Fig. 3C).

A total of six tumors (85.7%) were completely solid (Figs. 1, 2, 3, 4) with an average diameter of 5.28 cm, and one tumor showed mixed solid and cystic characteristics. No significant central necrosis was found. Calcification (Fig. 2A) was observed in one mass (14.3%). One patient exhibited hemorrhage, which presented as a high-density region on nonenhanced CT scans. Three tumors (42.9%) had thin, well-circumscribed, enhanced encapsulation (Fig. 2C). One patient exhibited a pancreatic head tumor with mild dilatation of the pancreatic common duct, and no patients suffered from pancreatic atrophy. Two tumors showed isointensity on T1-weighted imaging (T1WI) (Fig. 1A), while on T2-weighted imaging (T2WI), one tumor showed isointensity (Fig. 3B), and one showed hypointensity (Fig. 1C).

Compared with the adjacent normal pancreatic parenchyma on the noncontrast CT scan, six tumors (85.7%) showed isodensity (Fig. 2A), while one (14.3%) showed hypodensity. All tumors showed hypodensity in the AP (Fig. 3A). Four tumors (57.1%) showed hypodensity (Fig. 1D), while the other three (42.9%) showed isodensity in the VP (Figs. 2C, 4A). Six small tumors (85.7%), with an average diameter of 5.28 cm, showed homogeneous enhancement (Fig. 1D), while the large lesions showed nonhomogeneous and septal enhancement due to cystic necrosis; none of the tumors displayed ring enhancement. With dynamic contrast-enhanced MRI, all tumors (n = 2) showed hypointensity in the AP and portal phase (Fig. 1C), isointensity in the VP (Fig. 3C) as well as progressive enhancement.

**Figure 5:**
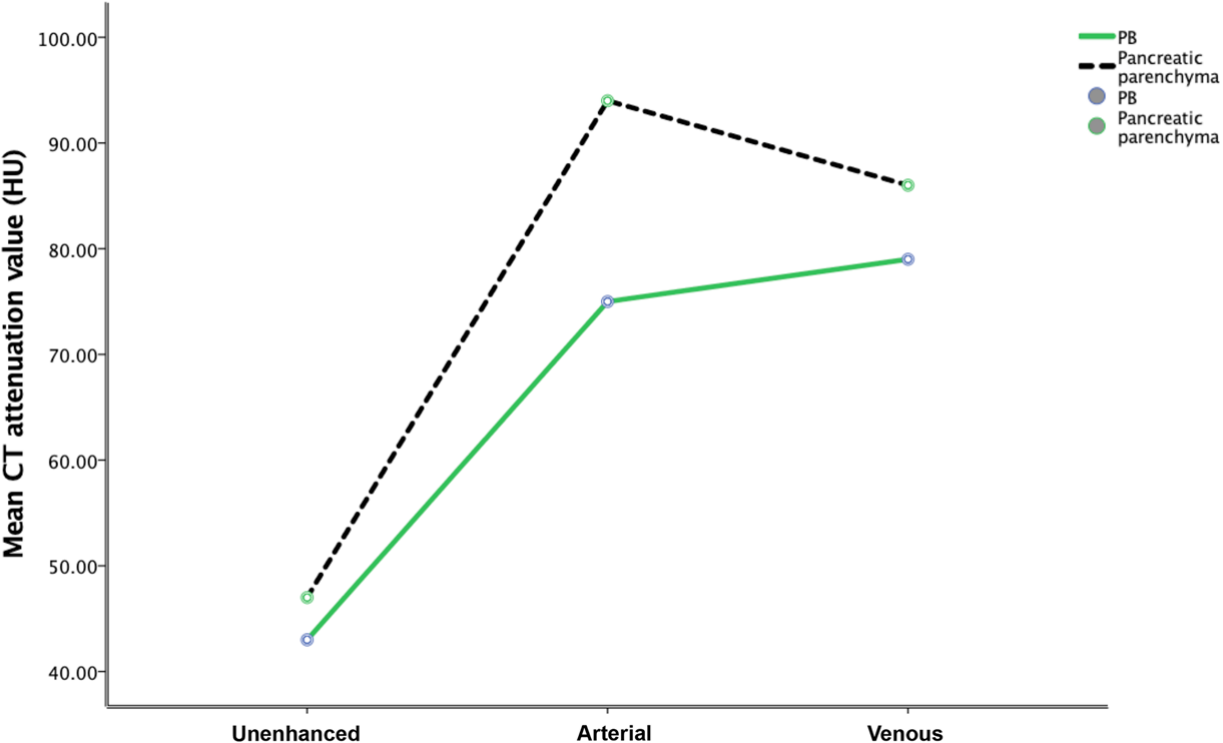
Mean CT attenuation values of the tumors and the surrounding pancreatic parenchyma in the three phases. In the AP, the attenuation value of the tumors was significantly lower than that of the surrounding normal pancreatic parenchyma (P < 0.001), while in the unenhanced phase and VP, the attenuation value of the tumors was slightly lower than that of the surrounding normal pancreatic parenchyma (P = 0.193 and P = 0.049, respectively).

PB is a hypovascular tumor that displays a gradually increasing pattern in noncontrast, AP, and VP CT scans (Fig. 5). The CT attenuation value of the tumor in the AP was significantly higher than that in the noncontrast phase (P < 0.001), and the CT attenuation value of the tumor in the VP was slightly higher than that in the AP, but this difference was not significant (P = 0.877). In the five patients with triphasic CT scans, the TAC showed that peak enhancement of the tumors occurred in the VP, while in the other two patients with quadriphasic CT scans, the TAC showed that the peak enhancement of the tumors occurred in the portal phase and that enhancement slightly decreased in the VP.

The TAC in Fig. 5 shows that in all three phases (noncontrast, arterial, and venous phases), the CT attenuation values of the pancreatic parenchyma were higher than those of the PB. In the AP, the CT attenuation values of the tumor were significantly lower than those of the normal pancreatic parenchyma (P < 0.001), and the tumor was also the most clearly visualized. In the unenhanced phase and VP phase, the CT attenuation values of the tumor were slightly lower than those of the pancreatic parenchyma (P = 0.193 and P = 0.049, respectively).

^18^F-FDG PET/CT imaging showed no uptake in two pancreatic tumors (Fig. 1E); one of the pancreatic tumors was associated with multiple liver metastases, but only one metastatic lesion showed mild focal uptake (Fig. 1E). The third patient arrived at our hospital for surgical treatment after a PET/CT scan conducted at another hospital revealed a pancreatic head mass accompanied by increased FDG uptake (SUVmax = 4.22).

### Pathological features

**Figure 6:**
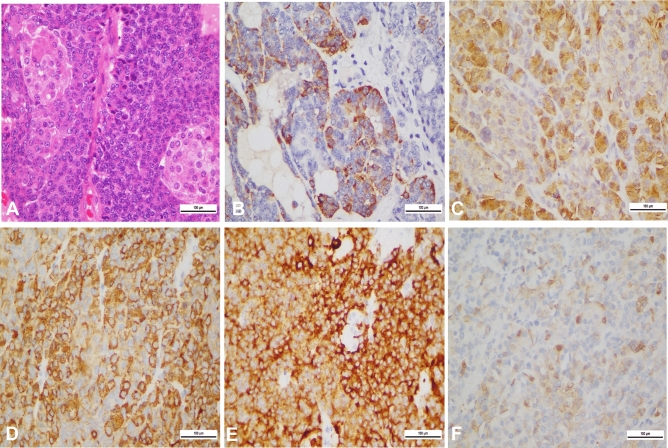
Hematoxylin–eosin staining and immunohistochemistry revealed the squamous corpuscle and multidifferentiation of PB (same patient as Fig. 1). (**A**) Squamoid nest-like island of epithelioid. (**B**) Immunolabeling for trypsin. (**C**) Immunolabeling for chymotrypsin. (**D**) Immunolabeling for chromogranin. (**E**) Immunolabeling for synaptophysin. (**F**) Immunolabeling for trypsin. (**F**) Immunolabeling for β-catenin. Note the mosaic pattern of the membranous and nuclear labeling.

Grossly, the tumors were soft and well circumscribed, the excised sections of the tumor were gray-white with clear margins, and three tumors were encapsulated. The relative proportions of solid and cystic areas correlated well with the CT and MRI findings. Microscopically, for all seven cases, epithelial elements of PB are highly cellular and arranged in well-defined islands separated by stromal bands, producing a geographic low-power appearance. The squamoid nests were observed in all cases with biotin-rich, optically clear nuclei (Fig. 6A). Cells with acinar differentiation and neuroendocrine differentiation were identified by immunohistochemistry. For all seven cases, immunolabelling was remarkable, showing diffuse or patchy labelling for acinar markers [trypsin (Fig. 6B), chymotrypsin (Fig. 6C)] and neuroendocrine markers [chromogranin (Fig. 6D) and synaptophysin (Fig. 6E)]. In five cases, patchy or limited nuclear and cytoplasmic immunolabelling for β-catenin was observed (Fig. 6F).

### Clinical follow-up

While only a single patient demonstrated liver metastasis on their initial MRI, three other patients developed liver metastases—at 1, 4, and 12 months after surgery.

A review of the clinical records revealed that two patients remained alive at the time of the writing of this article, having survived between 189 and 719 days. Four patients were lost to follow-up at 47, 266, 299 and 1,443 days after surgery. The remaining patient underwent surgery after 6 months of neoadjuvant chemotherapy and was lost to follow-up at 239 days after surgery.

## Discussion

Because of the rarity of this tumor, only individual cases of PB have been reported in the clinical or imaging literature, and most of our understanding of the imaging characteristics of adult PB is based on these case reports^5,9–16^. To the best of our knowledge, we believe that our series of seven patients is the largest of its kind in the radiology literature.

The clinical presentation of PB is often nonspecific. The most common presenting symptom in our series was abdominal pain (42.9%), which is similar to the findings of prior reports^9^. According to the literature, the incidence of PB in males is slightly higher than that in females^2,9,17^. However, in our study, all seven patients were male, which may have been due to the relatively small sample size. Elevated alpha-fetoprotein (AFP) expression has been reported in nearly 68% of pediatric PB patients^18,19^. In contrast, our study reveals that the vast majority of adult patients usually have tumor markers within normal ranges.

Our study and the literature show that PB is soft, well demarcated and often encapsulated based on pathological analyses^18,20–23^, which may explain why PB rarely causes pancreatic duct dilatation^22^. In addition, adult PB contains less stroma than pediatric PB^23^. In our study, all the tumors were exogenous, and tumors grew outward from the pancreatic parenchyma, similar to branches growing from a tree trunk. We hypothesize that this finding may be due to the soft and noninfiltrating nature of the tumor, making it easier for the tumor to grow into the low-resistance fat spaces around the pancreas. To the best of our knowledge, this imaging presentation has not been reported in pediatric patients with PB or in populations with other pancreatic tumors.

Horie et al.^24,25^ subdivided PB into tumors that arise from the ventral anlage (right-sided tumors: head of the pancreas) and those that arise from the dorsal anlage (left-sided tumors: body and tail of the pancreas). Many reports on childhood PB have suggested that ventral anlage tumors are usually well encapsulated, do not show calcification and generally have a good prognosis, whereas dorsal anlage tumors are not encapsulated, exhibit calcification and have a more insidious clinical onset along with a relatively poor prognosis^24,26^. However, in our study of adult PB, only one of the three patients with right-sided tumors showed incomplete encapsulation, while the other two patients did not present with tumor encapsulation or their tumors were ill defined. Of these two cases, one involved the duodenum and peripheral blood vessels, while the other involved multiple liver metastases. No calcification was observed in any of the three patients with right-sided tumors. In contrast, in the four patients with left-sided tumors, two showed encapsulation, all four had clear margins, and none had metastasis or peripheral invasion at the initial diagnosis. These results indicate that right-sided tumors are more aggressive than left-sided tumors. This may be because most of the previous studies focused on pediatric cases, while our study focused on adult cases, and the biological characteristics of children's cases and adult cases may differ^9^.

Montemarano et al. suggested that the majority of PBs are large, well-defined, heterogeneous masses that are at least partially circumscribed^17^. Other studies have reported that PB can present with obvious necrosis or even complete cystic degeneration^27–29^. However, in our study, six tumors (85.7%) were purely solid and homogeneous, which may be related to the small mean diameter of the included tumors. Limited published data are available regarding the enhancement pattern of PB. In our study, PB was observed to be a hypovascular neoplasm with progressive enhancement. However, very few authors have demonstrated that PB is hypervascular in the pancreatic phase and then isodense in the PVP^15^, perhaps because there are more neuroendocrine differentiation components in these tumors.

To the best of our knowledge, there are no reports on ^18^F-FDG PET/CT imaging of adult PB. ^18^F-FDG PET/CT imaging of PB in children has been rarely reported, and these sporadic reports have shown that the tumor and its metastases have varying degrees of uptake^30,31^. However, in our study, only one of the three adult PB tumors showed FDG uptake, while the other two tumors had no FDG uptake. Of these two patients, one had multiple liver metastases, and in this patient, only one of the liver metastases showed mild focal uptake on FDG PET/CT imaging. To the best of our knowledge, our observation that PB does not show uptake on ^18^F-FDG PET/CT imaging has not yet been reported in the literature.

The radiological differential diagnosis of PB includes nonfunctional neuroendocrine neoplasms, solid pseudopapillary neoplasms (SPNs) of the pancreas, and ACC. Most neuroendocrine neoplasms are located in the parenchyma of the pancreas and are significantly enhanced in the AP and reduced in the VP^32^. Typically, SPNs tend to occur in young women. However, in the literature, the number of adult male patients with SPNs is higher than the number of adults with PB^33^. SPNs in men presents as a somewhat large, solid mass with lobulated margins^34^.

Both ACC and PB are considered pancreatic tumors with acinar differentiation. These neoplasms are clinically, pathologically, and genetically unique when compared to more common pancreatic neoplasms. The average age of onset for ACC has been reported to be approximately 60 years old, which is older than that for adult PB^35^.

There are several limitations in this investigation that should be noted. First, because of its retrospective nature, this study may have selection and verification bias. Second, the sample size is relatively small because adult PB is extremely rare.

In conclusion, adult PB most commonly occurs in male patients and has obvious imaging features, often presenting as a solid mass with well-defined boundaries and encapsulation. PB typically exhibits an exophytic growth pattern, similar to branches growing outward from a tree trunk. After injection of a contrast agent, the lesions present as hypovascularized masses with homogeneous and progressive enhancement on CT and MRI. PB does not usually show uptake on ^18^F-PET/CT.

## Data Availability

The datasets generated during and/or analysed during the current study are available from the corresponding author on reasonable request.

## Abbreviations

PB: Pancreatoblastoma
ROI: Region of interest
TAC: Time-density curve
SUVmax: Maximum standardized uptake value
SPN: Solid pseudopapillary neoplasm
ACC: Acinar cell carcinoma
